# A non-rigid map fusion-based direct SLAM method for endoscopic capsule robots

**DOI:** 10.1007/s41315-017-0036-4

**Published:** 2017-11-24

**Authors:** Mehmet Turan, Yasin Almalioglu, Helder Araujo, Ender Konukoglu, Metin Sitti

**Affiliations:** 1 Max Planck Institute for Intelligent Systems, Stuttgart, Germany; 2 Computer Vision Laboratory, ETH Zurich, Zurich, Switzerland; 3 Robotics Laboratory, University of Coimbra, Coimbra, Portugal

**Keywords:** Endoscopic capsule robot, Dense direct medical SLAM, Non-rigid frame-to-model fusion

## Abstract

Since the development of capsule endoscopy technology, medical device companies and research groups have made significant progress to turn passive capsule endoscopes into robotic active capsule endoscopes. However, the use of robotic capsules in endoscopy still has some challenges. One such challenge is the precise localization of the actively controlled robot in real-time. In this paper, we propose a non-rigid map fusion based direct simultaneous localization and mapping method for endoscopic capsule robots. The proposed method achieves high accuracy for extensive evaluations of pose estimation and map reconstruction performed on a non-rigid, realistic surgical EsophagoGastroDuodenoscopy Simulator and outperforms state-of-the art methods.

## Introduction

In the past decade, advances in microsensors and microelectronics have enabled small, low cost devices in a variety of high impact applications. Following these advances, untethered pill-size, swallowable capsule endoscopes with an on-board camera and wireless image transmission device have been developed and used in hospitals for screening the gastrointestinal (GI) tract and diagnosing diseases such as the inflammatory bowel disease, the ulcerative colitis, and the colorectal cancer. Unlike standard endoscopy, endoscopic capsule robots are non-invasive, painless, and more appropriate to be employed for long-duration screening purposes. Moreover, they can access difficult body parts that were not possible to reach before with standard endoscopy (e.g., small intestines). Such advantages make pill-size capsule endoscopes a significant alternative screening method over standard endoscopy (Liao et al. 2010; Nakamura et al. 2008; Pan and Wang 2012; Than et al. 2012). However, current capsule endoscopes used in hospitals are passive devices controlled by peristaltic motions of the inner organs. The control over capsule’s position, orientation, and functions would give the doctor a more precise reachability of targeted body parts and more intuitive and correct diagnosis opportunity. Several groups have recently proposed active, remotely controllable robotic capsule endoscope prototypes equipped with additional functionalities, such as local drug delivery, biopsy, and other medical functions (Sitti et al. 2015; Yim et al. 2013; Carpi et al. 2011; Keller et al. 2012; Mahoney et al. 2013; Yim et al. 2014). An active motion control is, on the other hand, heavily dependent on a precise and reliable real-time pose estimation capability, which makes the robot localization and mapping the key capability for a successful endoscopic capsule robot operation. Localization methods such as (Fluckiger and Nelson 2007; Rubin et al. 2006; Kim et al. 2008; Son et al. 2016) have the common drawback that they require extra sensors and hardware to be integrated to the robotic capsule system. Such extra sensors have their own drawbacks and limitations if it comes to their application in small-scale medical devices, e.g. space limitations, cost aspects, design incompatibilities, biocompatibility issues, and most importantly the interference of the sensors with the activation system of the capsule robot.

**Fig. 1:**
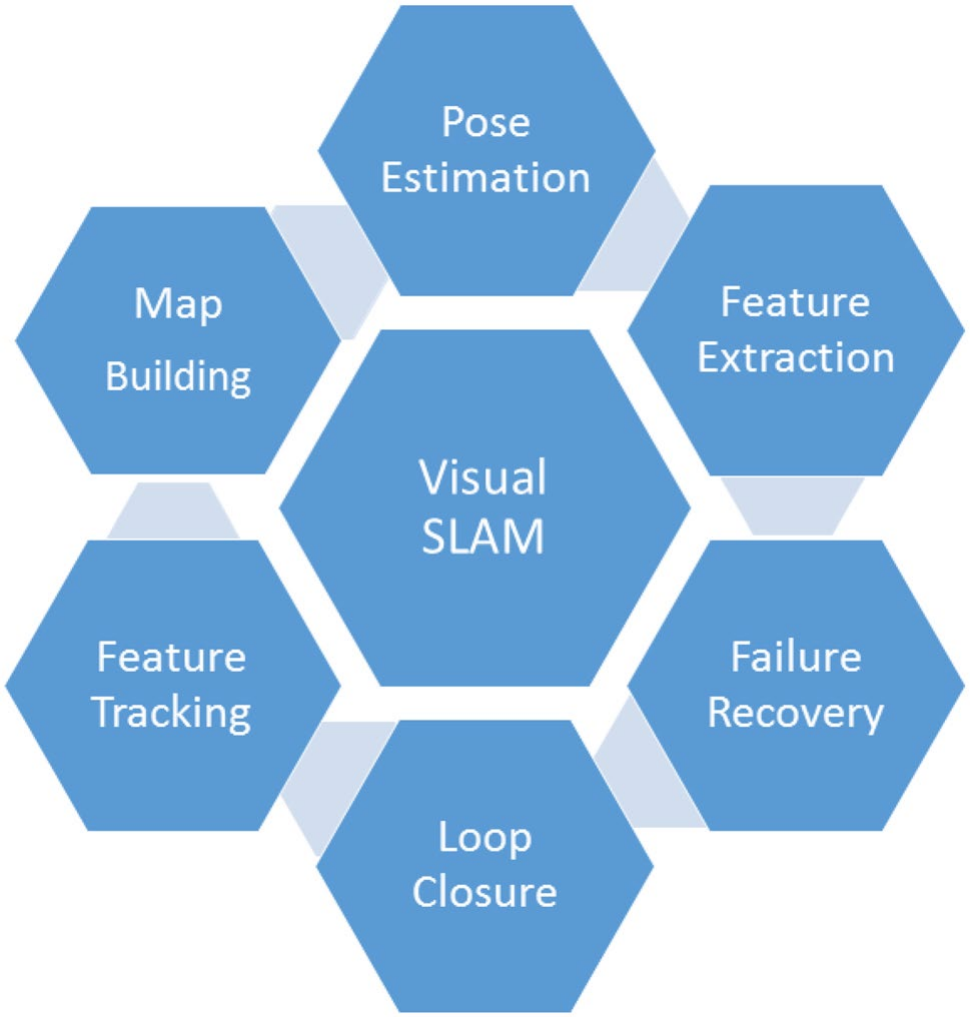
Components of a modern vSLAM

As a solution of these issues, vision-based localization and mapping methods (vSLAM) have attracted the attention for small-scale medical devices. With their low cost and small size, cameras are frequently used in localization applications where weight and power consumption are limiting factors, such as in the case of small-scale robots. However, many challenges posed by the GI tract and low quality cameras of the endoscopic capsule robots cause further difficulties in front of a vSLAM technique to be applied in a medical operation. Self-repetitiveness of the GI tract texture, non-rigid organ deformations, heavy reflections caused by the organ fluids, and lack of distinctive feature points on the GI tract tissue are further challenges in front of a reliable robotic operation. Moreover, the low frame rate and limited resolution of the current capsule camera systems also restrict the applicability of computer vision methods inside the GI tract. Especially feature tracking based visual localization methods have poor performance in the abdomen region compared to outdoor or indoor large scale environments where unique features can be found easier.

Figure 1 gives an overview of a modern vSLAM approach with its key components. A modern vSLAM method is expected to be equipped with reliable pose estimation and map reconstruction modules that is not affected by non-rigid deformations, sudden frame-to-frame movements, blur, noise, illumination changes, occlusions and large depth variations. Moreover, dynamic structure of the GI tract organs with heavy peristaltic motions require more than a static map; reconstructed parts of the map must be updated continuously as the organ structure changes during endoscopic operation. Besides, a failure recovery procedure relocalizing the robot after unexpected drifts is a further demand on a modern vSLAM system. The intra-operative 3D reconstruction of the explored inner organ simultaneous to tracking capsule robot position in real-time provides key information for the next generation actively controllable endoscopic robots which will be equipped with functionalities such as disease detection, local drug delivery and biopsy. Feature- based SLAM methods have been applied on endoscopic type of videos in the past decades (Mountney and Yang 2009; Casado et al. 2014; Stoyanov et al. 2010; Mountney and Yang 2010; Mountney et al. 2006; Qian et al. 2013; Mahmoud et al. 2016). However, besides sparse unrealistic map reconstruction, all of these methods suffer from heavy drifts and inaccurate pose estimations once low texture areas are entered. With that motivation, we developed a direct medical vSLAM method which shows high accuracy in terms of map reconstruction and pose estimation inside GI tract.

## Method

In that section, we first summarize the contributions of our paper and give details of the proposed method.

### Contributions of the method

**Fig. 2:**
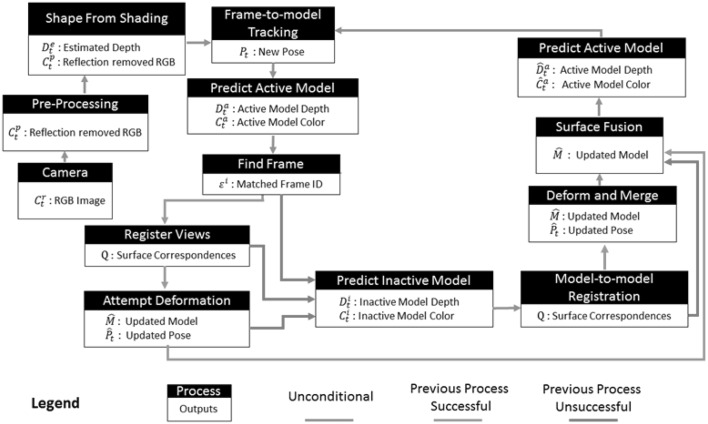
Overview of the proposed medical SLAM method

Inspired from large-scale RGB Depth SLAM approaches (Whelan et al. 2015; Newcombe et al. 2011), the proposed method is to the best of our knowledge the first fully dense, direct medical SLAM approach using GPU accelerated non-rigid frame-to-model fusion, joint volumetric-photometric pose estimation and dense model-to-model loop closure techniques. Figure 2 depicts the system architecture diagram and below the key steps of the proposed framework are summarized:

- Create depth image from RGB image based on shading;
- Divide visited organ parts into active and inactive areas. Only active areas are used for pose tracking and map fusion. Areas that do not appear in the scene for a certain period of time are assigned as inactive and not used in the estimation.
- For every new frame, search for its intersection with the active model and fuse them;
- In case there exists an intersection of the active model with inactive model within the current frame, fuse the intersecting parts using loop closure and reactivate corresponding inactive parts.

The contributions of the approach described in this paper include:

- A vSLAM approach able to deal with specularities typically occurring in images of inner organs tissues;
- A direct vSLAM method able to handle non-rigid structures, including performing their non-sparse 3D reconstruction;
- A direct vSLAM approach jointly minimizing photometric-geometric constraints, including depth;

### Preprocessing and depth image creation

**Fig. 3:**
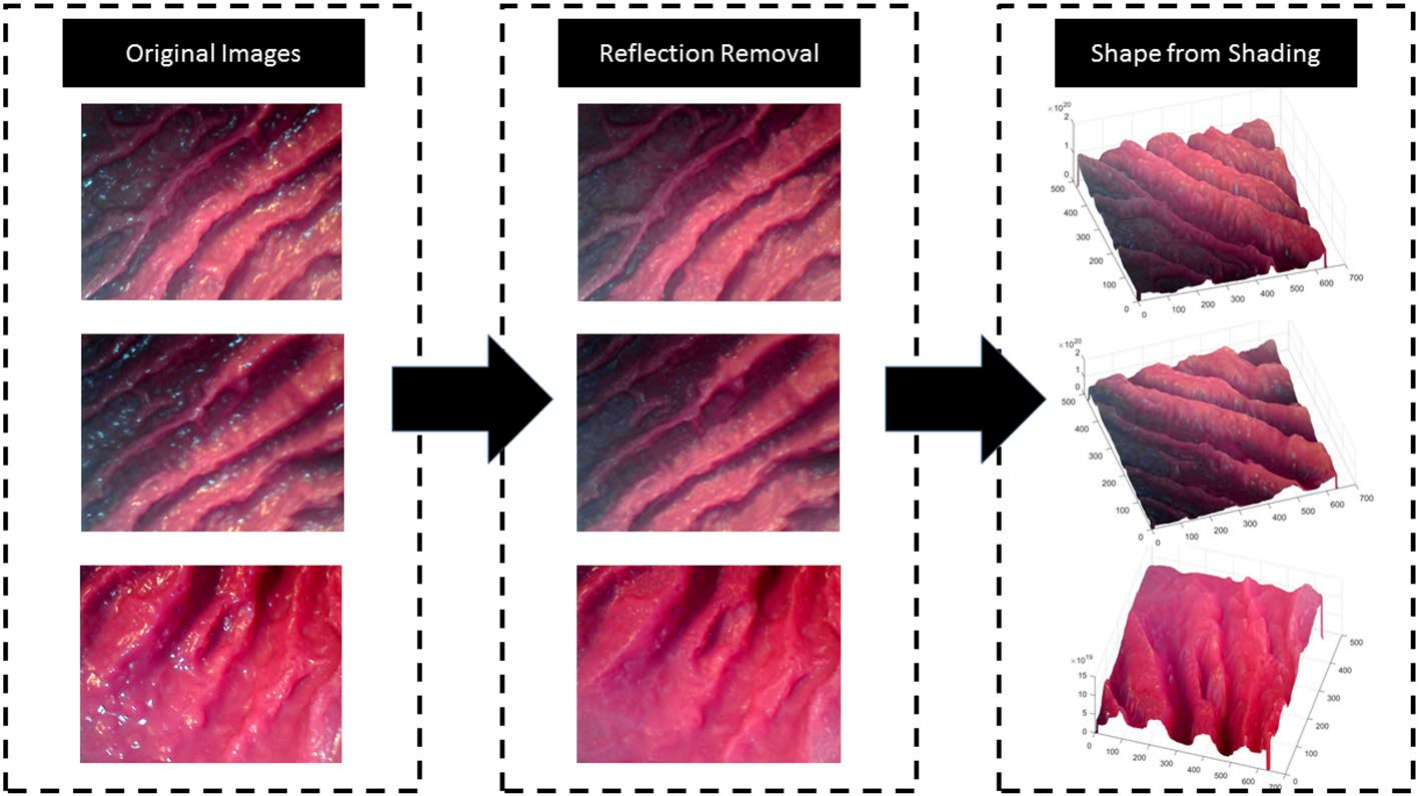
Reflection suppression and shading-based depth image creation

The framework starts with a preprocessing module that suppresses specularities caused by inner organ fluids. Reflection detection is done by combining the gradient map of the input image with the peak values detected by an adaptive threshold. Once specularities detected, suppression is performed by inpainting. Next, GPU accelerated version of Tsai-Shah shading method is applied to create depth images. This method uses linear approximations to extract depth image from RGB input iteratively estimating slant, tilt and albedo values. For further details, the reader is referred to the original paper (Ping-Sing and Shah 1994). Figure 3 demonstrates examples of input RGB images, images after reflection suppression and depth images acquired by Tsai-Shah shading method.

### Joint photometric and geometric pose estimation from a splattered surfel prediction

The input for pose estimation is the RGB image $\mathcal {C}$ and the depth image $\mathcal {D}$. We combine photometric and geometric pose estimation techniques. The camera pose of the endoscopic capsule robot is described by a transformation matrix $\mathbf P _t$:

1 $$\begin{aligned} \mathbf P _t = \begin{bmatrix}&\mathbf R _t&\mathbf t _t\\ 0&0&0&1 \end{bmatrix} \in \mathbb {SE}_3. \end{aligned}$$

Given the depth image $\mathcal {D}$, the 3D back-projection of a point $\mathbf u$ is defined as ${\mathbf{p}}({\mathbf{u}},\mathcal {D})$ = **K** $^{-1}$ ***u***d(**u**), where **K** is the camera intrinsics matrix and ***u*** is the homogeneous form of **u**. Geometric pose estimation is performed by minimizing the energy cost function $E_{icp}$ between the current depth image $\mathcal {D}^l_t$ and the active depth model $\hat{\mathcal {D}}_{t-1}^a$:

2 $$\begin{aligned} E_{icp} = \sum _k{((\mathbf v ^k - \exp {(\hat{\xi })}{} \mathbf T \mathbf v _k^t)\cdot \mathbf n ^k)^2} \end{aligned}$$

where $\mathbf v ^k_t$ is the back-projection of the *k*-th vertex in $\mathcal {D}^l_t$, $\mathbf v ^k$ and $\mathbf n ^k$ are the corresponding vertex and normal from the previous frame. Thus, $\mathbf T$ is the estimated transformation from the previous to current robot pose and $\exp {(\hat{\xi })}$ is the exponential mapping function from Lie algebra $\mathfrak {se}_3$ to Lie group $\mathbb {SE}_3$. Analogously, the photometric pose $\xi$ between the current RGB image $\mathcal {C}^l_t$ and active RGB model $\hat{\mathcal {C}}^a_{t-1}$ is estimated by minimizing photometric energy cost function:

3 $$\begin{aligned} E_{rgb} = \sum _\mathbf{u \in \Omega } \left( I(\mathbf u ,\mathcal {C}^l_t) - I(\pi (\mathbf K \exp (\hat{\xi }) \mathbf T \mathbf p (\mathbf u , \mathcal {D}^l_t)), \hat{\mathcal {C}}_{t-1}^a) \right) ^2 \end{aligned}$$

The energy minimization function for joint photometric-geometric pose estimation is defined by:

4 $$\begin{aligned} E_\text {track} = E_\text {icp} + w_\text {rgb}E_\text {rgb}, \end{aligned}$$

which is minimized using Gauss–Newton non-linear least-squares optimization.

### Scene representation, deformation graph and loop closure

Due to strict real-time concerns of the approach, we use surfel-based scene reconstruction. Each surfel has a position, normal, color, weight, radius, initialization timestamp and last updated timestamp. We also define a deformation graph consisting of a set of nodes and edges to detect non-rigid deformations throughout the frame sequence. Each node $\mathcal {G}^n$ has a timestamp $\mathcal {G}^n_{t_0}$, a position $\mathcal {G}_g^n \in \mathbb {R}^3$ and a set of neighboring nodes $\mathcal {N}(\mathcal {G}^n$). The directed edges of the graph are neighbors of each node. A graph is connected up to a neighbor count *k* such that $\forall n,|\mathcal {N}(\mathcal {G}^n)| = k$. Each node also stores an affine transformation in the form of a $3\times 3$ matrix $\mathcal {G}^n_\mathbf{R }$ and a $3\times 1$ vector $\mathcal {G}_\mathbf{t }^n$. When deforming a surface, the $\mathcal {G}^n_\mathbf{R }$ and $\mathcal {G}_\mathbf{t }^n$ parameters of each node are optimized according to surface constraints. In order to apply a deformation graph to the surface, each surfel $\mathcal {M}^s$ identifies a set of influencing nodes in the graph $\mathcal {I}(\mathcal {M}^s, \mathcal {G})$. The deformed position of a surfel is given by:

5 $$\begin{aligned} \hat{\mathcal {M}}^s_\mathbf{p } = \phi (\mathcal {M}^s) = \sum _{n \in \mathcal {I}(\mathcal {M}^s, \mathcal {G})} w^n(\mathcal {M}^s) [\mathcal {G}^n_\mathbf{R }(\mathcal {M}_\mathbf{p }^s - \mathcal {G}_\mathbf{g }^n) + \mathcal {G}_\mathbf{g }^n + \mathcal {G}_\mathbf{t }^n] \end{aligned}$$

while the deformed normal of a surfel is given by:

6 $$\begin{aligned} \hat{\mathcal {M}}^s_\mathbf{p } = \sum _{n \in \mathcal {I}(\mathcal {M}^s, \mathcal {G})} w^n (\mathcal {M}^s)\mathcal {G}^{{n-1}^T}_\mathbf{R } \mathcal {M}^s_\mathbf{n }, \end{aligned}$$

where $w^n (\mathcal {M}^s)$ is a scalar representing the influence of $\mathcal {G}^n$ on surfel $\mathcal {M}^s$, summing to a total of 1 when $n = k$:

7 $$\begin{aligned} w^n (\mathcal {M}^s) = (1 - ||\mathcal {M}^s_\mathbf{p }-\mathcal {G}_\mathbf{g }^n ||_2 / d_\text {max})^2. \end{aligned}$$

Here, $d_\text {max}$ is the Euclidean distance to the $k+1$-nearest node of $M^s$.

To ensure a globally consistent surface reconstruction, the framework closes loops with the existing map as those areas are revisited. This loop closure is performed by fusing reactivated parts of the inactive model into the active model and simultaneously deactivating surfels which have not appeared for a period of time.

## Experiments and results

We evaluate the performance of our system both quantitatively and qualitatively in terms of trajectory estimation, surface reconstruction and computational performance.

### Dataset and equipment

**Fig. 4:**
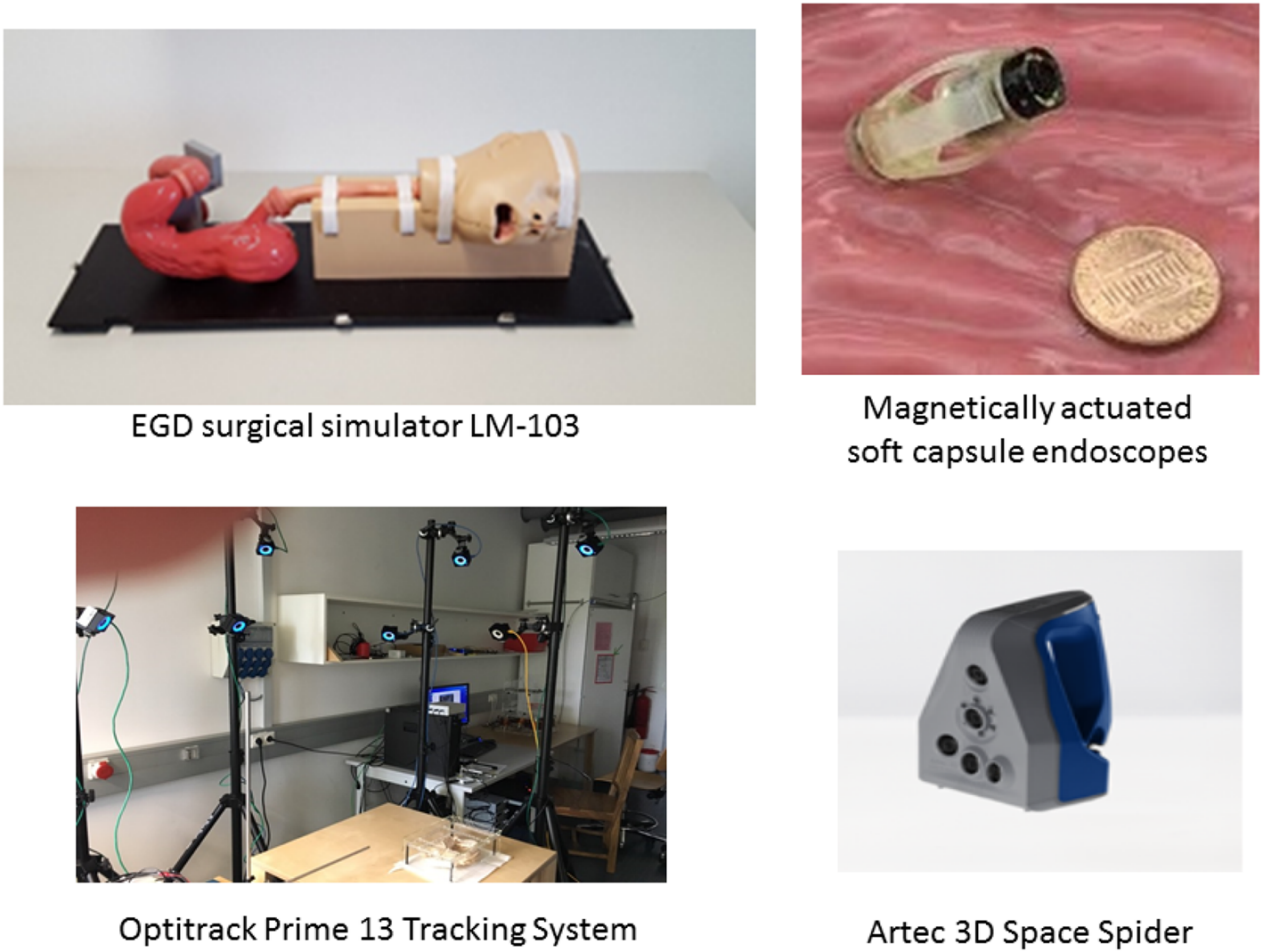
Experimental setup

**Fig. 5:**
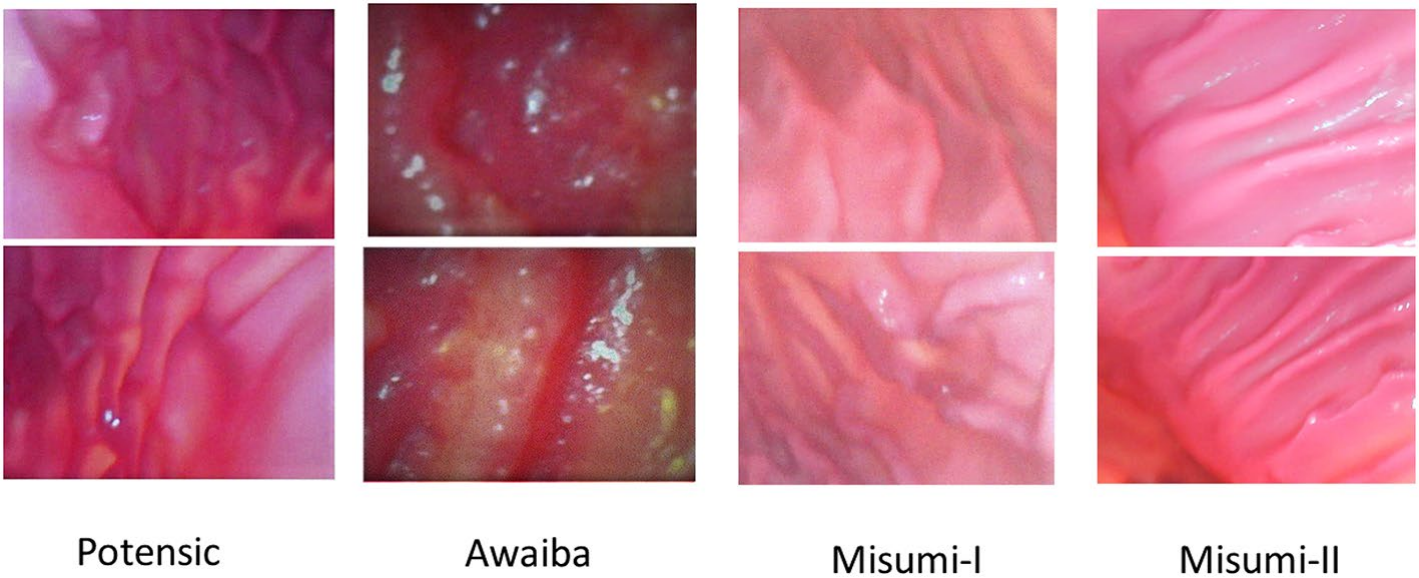
Sample images from our dataset

**Fig. 6:**
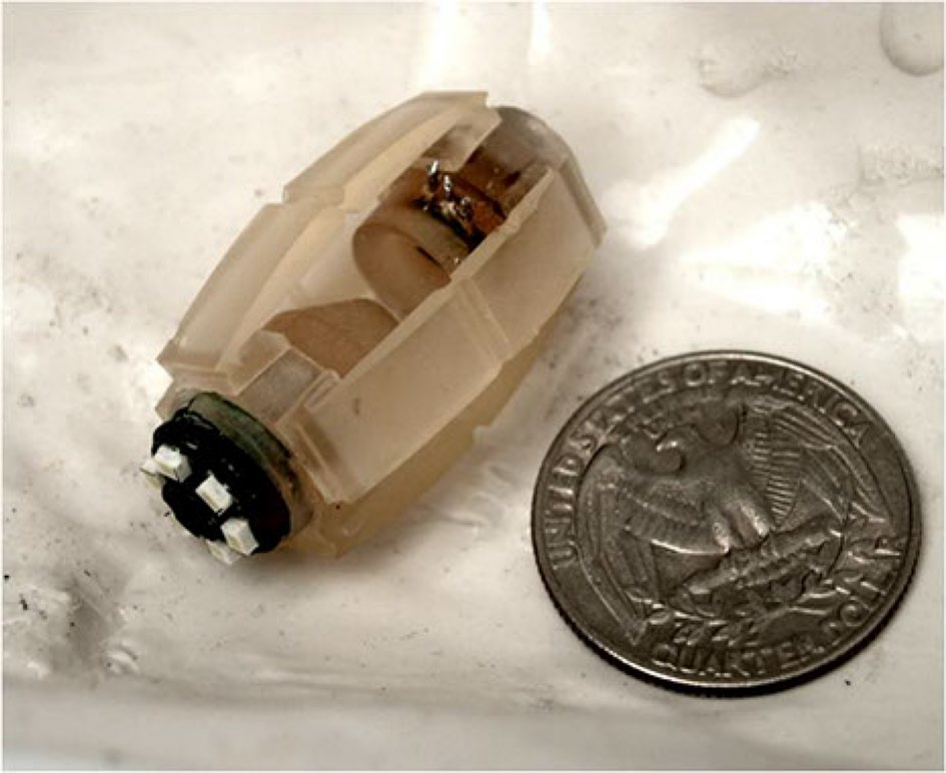
Photo of the endoscopic capsule robot prototype used in the experiments

Figure 4 shows our experimental setup as a visual reference. We created our own endoscopic capsule robot dataset with ground truth. To make sure that our dataset is general and does not lead to overfitting, three different endoscopic cameras were used to capture the endoscopic videos. We mounted endoscopic cameras on our magnetically activated soft capsule endoscope (MASCE) systems as seen in Fig. 6. The videos were recorded from an oiled non-rigid, surgical stomach model Koken LM103—EDG (EsophagoGastroDuodenoscopy) Simulator. Some sample frames are shown in Fig. 5. To obtain 6-DoF localization ground truth, an OptiTrack motion tracking system consisting of eight infrared cameras and a tracking software was utilized. A total of 15 minutes of stomach videos was recorded containing over 10,000 frames. Finally, we scanned the open surgical stomach model using a 3D Artec Space Spider image scanner. This scan served as the ground truth for the quantitative evaluations of the 3D map reconstruction module.

### Trajectory estimation

**Fig. 7:**
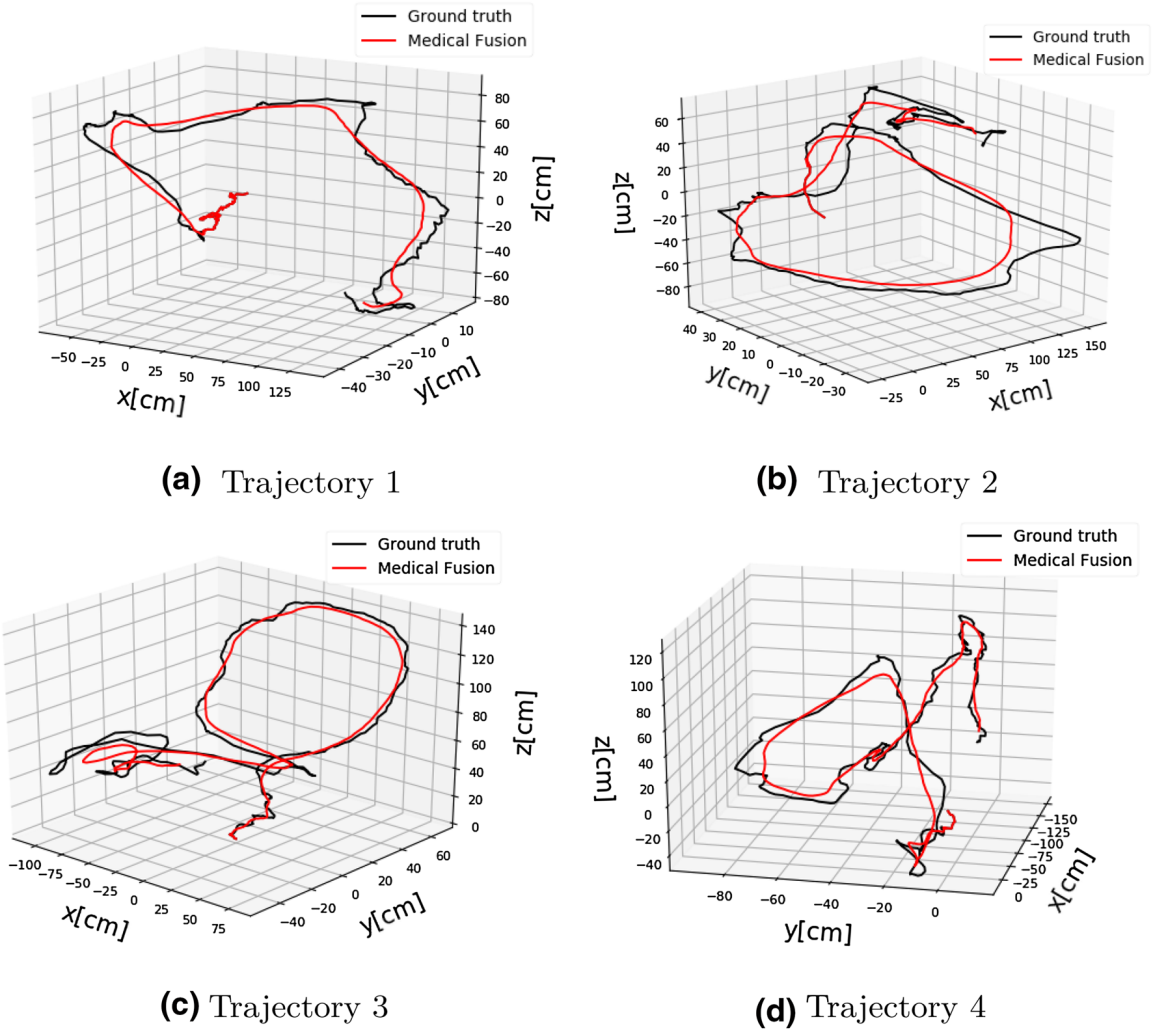
Sample trajectories estimated by the proposed method, ORB SLAM and ground truth

Table 1 demonstrates the results of the trajectory estimation for 7 different trajectories. The characteristics of the trajectories are as follows:

- Trajectory 1 is an uncomplicated path with slow incremental translations and rotations.
- Trajectory 2 follows a comprehensive scan of the stomach with many local loop closures.
- Trajectory 3 contains an extensive scan of the stomach with more complicated local loop closures.
- Trajectory 4 consists of more challenging motions including faster rotational and translational movements.
- Trajectory 5 consists of very loopy and complex motions.
- Trajectory 6 is the same as trajectory 5 but included added synthetic noise to allow checking the robustness of the system against noise.
- Before capturing trajectory 7, we added more paraffin oil into the simulator tissue to have stronger reflections. Similarly to trajectory 6, trajectory 7 consists of very loopy and complex motions including very fast rotations, translations and drifting.

Qualitative tracking results of the proposed direct medical SLAM compared to ORB SLAM and to ground truth are shown in Fig. 7. It is clearly observable that direct medical SLAM stays close to the ground truth except for minor deviations in loopy sections, whereas ORB SLAM has major deviations in many sections of the trajectories. For the quantitative analysis, we measured the root-mean-square of the Euclidean distances between the estimated camera poses and the ground truth. As seen in Table 1, the system performs very robustly and tracking accurately in all of the trajectories, not being affected by sudden movements, blur, noise or strong spectral reflections. Figure 9a, b represent rotational and translational RMSE results for different pose estimation strategies including frame-to-model alignment, photometric alignment, frame-to-frame alignment and ORB SLAM as a state-of-the art method. Results indicate that frame-to-model alignment clearly outperforms frame-to-frame alignment, photometric alignment and ORB SLAM. Besides, joint volumetric-photometric alignment outperforms photometric alignment indicating the significance of depth information for pose estimation. Figure 10a, b represent rotational and translational RMSE as a function of ICP weight in joint photometric-volumetric alignment (see Eq. 4). Both RMSEs decrease with higher ICP weights, reaching a minimum at $\omega = 87\%$ and $\omega = 85\%$, respectively.

**Table 1:** Trajectory lengths and RMSE results in meters for different endoscopic cameras

Trajectory ID	POTENSIC	MISUMI	AWAIBA	LENGTH
1	0.015	0.019	0.020	0.414
2	0.018	0.020	0.023	0.513
3	0.017	0.021	0.025	0.432
4	0.032	0.037	0.042	0.478
5	0.035	0.039	0.045	0.462
6	0.038	0.043	0.048	0.481
7	0.041	0.044	0.049	0.468

### Surface estimation

**Table 2:** Trajectory length and RMSE in meters for 3D surface reconstruction for different endoscopic cameras

Trajectory ID	POTENSIC	MISUMI	AWAIBA	Length
1	0.023	0.026	0.028	0.414
2	0.025	0.029	0.032	0.513
3	0.026	0.030	0.034	0.432
4	0.029	0.033	0.035	0.478
5	0.032	0.035	0.038	0.462
6	0.034	0.037	0.041	0.481
7	0.035	0.042	0.044	0.468

**Fig. 8:**
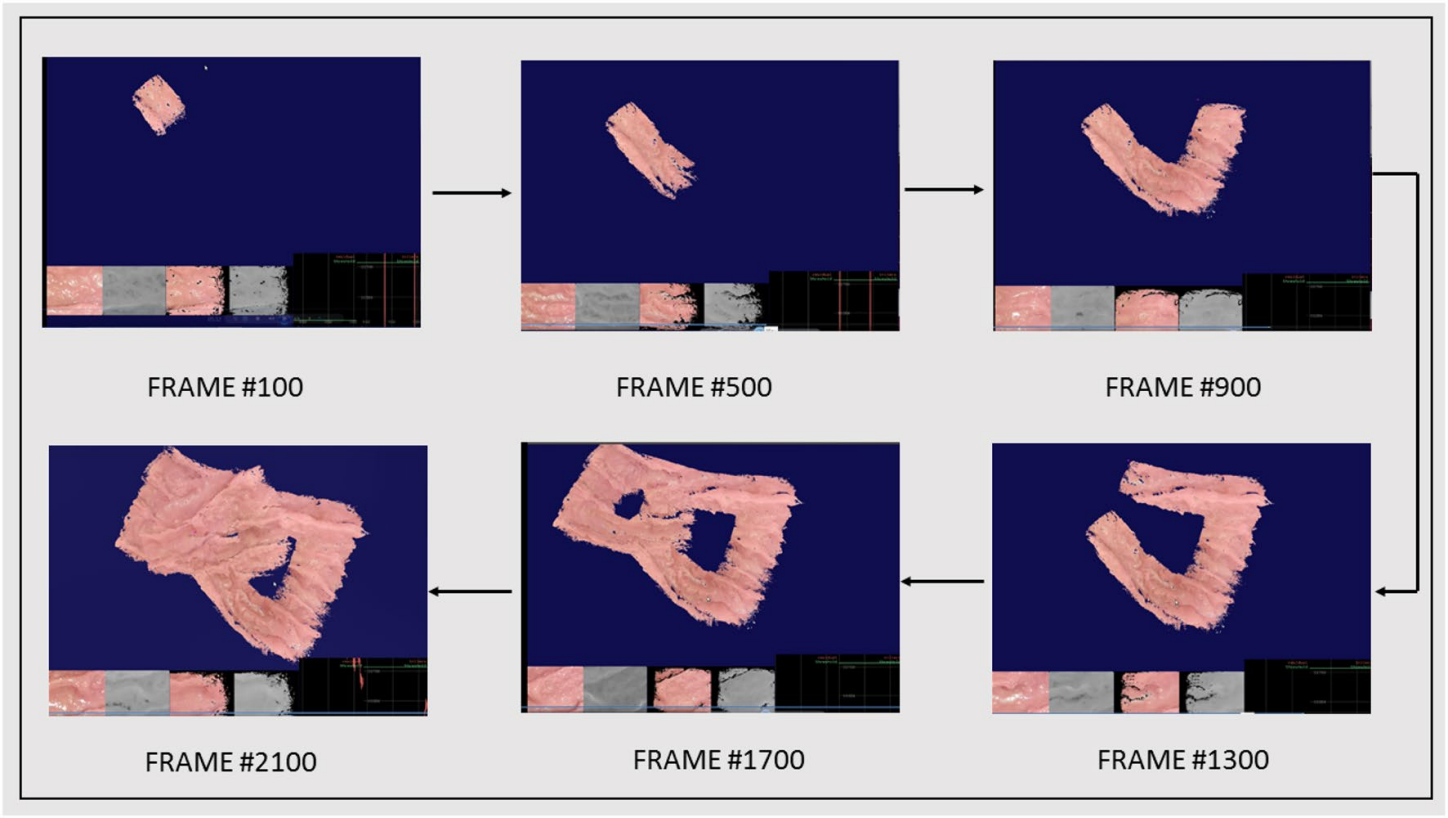
Frame-by-frame 3D reconstruction of the soft stomach simulator surface by the proposed medical SLAM method

We scanned the non-rigid EGD (Esophagogastroduodenoscopy) simulator to obtain the ground truth 3D data. Reconstructed 3D surface and ground truth 3D data were aligned using iterative closest point algorithm (ICP). RMSE for the reconstructed surface was calculated using the absolute trajectory (ATE) RMSE measuring the root-mean-square of the Euclidean distances between estimated depth values and the corresponding ground truth values. RMSE results in Table 2 show that even in very challenging trajectories with 4–7 sudden movements, strong noise and reflections, our system is capable of providing a reliable and accurate 3D surface reconstruction. A sample 3D reconstruction procedure is shown in Fig. 8 for visual reference.

**Fig. 9:**
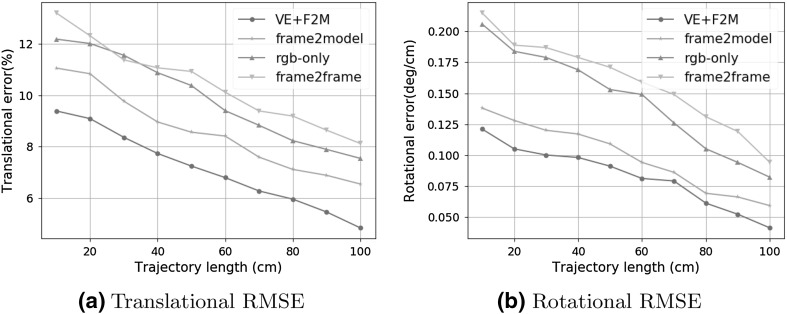
RMSE results for frame-to-model alignment (frame2model), photometric alignment (rgb-only) and frame-to-frame alignment (frame2frame)

**Fig. 10:**
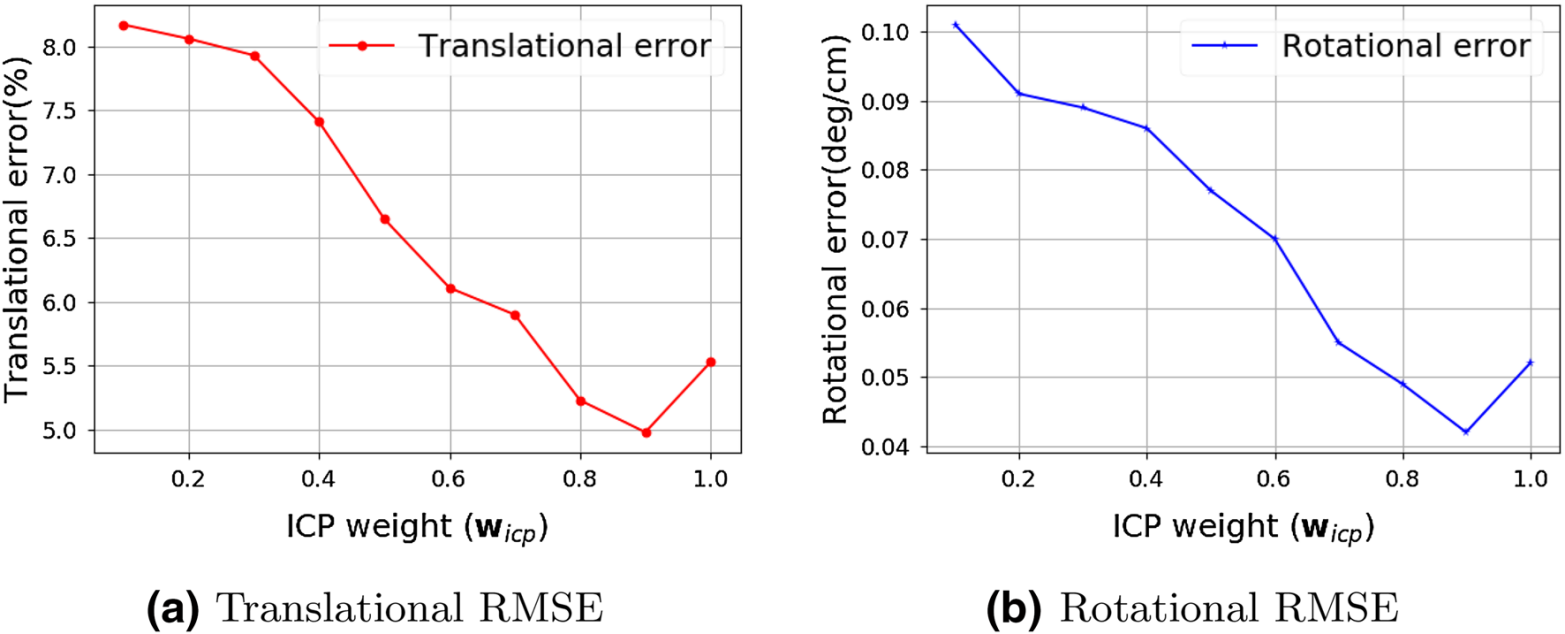
RMSE results vs ICP weight

### Computational performance

To analyze the computational performance of the system, we observed the average frame processing time across trajectories 1–4. The test platform was a desktop PC with an Intel Xeon E5-1660v3- CPU at 3.00, 8 cores, 32 GB of RAM and an NVIDIA Quadro K1200 GPU with 4 GB of memory. The execution time of the system depended on the number of surfels in the map, with an overall average of 48 ms per frame scaling to a peak average of 53 ms implying a worst case processing frequency of 18 Hz.

### Comparison with ORB SLAM

We compared the proposed method with ORB SLAM using our endoscopic capsule dataset. We chose ORB SLAM due to its state-of-the-art performance in various tasks, publicly available code and its recent use in endoscopic applications. We make the following observations after a detailed theoretical and practical evaluation of the differences between the proposed medical SLAM and ORB SLAM:

- ORB SLAM is based on feature matching while direct medical SLAM uses joint photometric- geometric pose estimation. In our evaluation, we observed that for endoscopic images, direct pose estimation is advantageous as compared to feature-based methods because specularity, noise and presence of fewer robustly identifiable features reduce the matching accuracy across frames.
- Direct medical SLAM needs a good initialization for pose estimation to avoid local minima while ORB SLAM does not require initialization.
- Direct medical SLAM employs a frame-to-model alignment strategy, which is robust to unexpected severe drifts inside GI tract. ORB SLAM on the other hand, performs frame-to-frame alignment and may have difficulties recovering from such drifts.
- Direct medical SLAM is computationally heavy while ORB SLAM can run on standard CPU in real-time. However, modern GPUs can be used to accelerate direct medical SLAM to near real-time as well.
- Direct medical SLAM tolerates larger motions between successive frames, while ORB SLAM expects smaller motions. However, we observed that both methods fail for very large inter-frame motion that leads to small overlap between successive frames.
- ORB SLAM’s reconstruction is in the form of a sparse point cloud of the scanned inner organ, whereas direct medical SLAM creates a dense and high quality 3D map of the organ.
- Qualitative and quantitative comparisons depicted in Figs. 7, 9a, b indicate large deviations of ORB SLAM from ground truth, whereas our method is able to stay close to the ground truth even in loopy parts of the trajectories.

## Conclusion

In this paper, we presented a direct and dense visual SLAM method for endoscopic capsule robots. Our system makes use of surfel-based dense data fusion in combination with frame-to-model tracking and non-rigid deformation. Experimental results suggest the effectiveness of the proposed system, both quantitatively and qualitatively, in occasionally looping endoscopic capsule robot trajectories and comprehensive inner organ scanning tasks. In future, we aim to extend our work into stereo capsule endoscopy applications to achieve even more accurate localization and mapping.

## References

[CR1] Carpi F, Kastelein N, Talcott M, Pappone C (2011). Magnetically controllable gastrointestinal steering of video capsules. IEEE Trans. Biomed. Eng..

[CR2] Casado S, Gil I, Montiel J, Grasa OG, Bernal E (2014). Visual slam for handheld monocular endoscope. Med. Imaging IEEE Trans..

[CR3] Fluckiger, M., Nelson, B.J.: Ultrasound emitter localization in heterogeneous media. In: 29th Annual International Conference of the IEEE Engineering in Medicine and Biology Society. IEEE **2007**, 2867–2870 (2007) 10.1109/IEMBS.2007.4352927 18002593

[CR4] Keller, H., Juloski, A., Kawano, H., Bechtold, M., Kimura, A., Takizawa, H., Kuth, R.: Method for navigation and control of a magnetically guided capsule endoscope in the human stomach. In: 2012 4th IEEE RAS & EMBS International Conference on Biomedical Robotics and Biomechatronics (BioRob), IEEE, pp. 859–865 (2012)

[CR5] Kim K, Johnson LA, Jia C, Joyce JC, Rangwalla S, Higgins PD, Rubin JM (2008). Noninvasive ultrasound elasticity imaging (uei) of crohn’s disease: animal model. Ultrasound Med. Biol..

[CR6] Liao Z, Gao R, Xu C, Li Z-S (2010). Indications and detection, completion, and retention rates of small-bowel capsule endoscopy: a systematic review. Gastrointest. Endosc..

[CR7] Mahmoud, N., Cirauqui, I., Hostettler, A., Doignon, C., Soler, L., Marescaux, J., Montiel, J.: Orbslam-based endoscope tracking and 3d reconstruction. arXiv preprint arXiv:1608.08149 (2016)

[CR8] Mahoney, A.W., Wright, S.E., Abbott, J.J.: Managing the attractive magnetic force between an untethered magnetically actuated tool and a rotating permanent magnet. In: Robotics and Automation (ICRA), 2013 IEEE International Conference on, IEEE, pp. 5366–5371 (2013)

[CR9] Mountney, P., Stoyanov, D., Davison, A., Yang, G.-Z.: Simultaneous stereoscope localization and soft-tissue mapping for minimal invasive surgery. In: International Conference on Medical Image Computing and Computer-Assisted Intervention, Springer, pp. 347–354 (2006) 10.1007/11866565_43 17354909

[CR10] Mountney, P., Yang, G.Z.: Dynamic view expansion for minimally invasive surgery using simultaneous localization, mapping, Visual slam for handheld monocular endoscope, Annual International Conference of the IEEE Engineering in Medicine and Biology Society (2009) 10.1109/IEMBS.2009.5333939 19964502

[CR11] Mountney, P., Yang, G.-Z.: Motion compensated slam for image guided surgery. Med. Image Comput. Comput. Assist. Intervent. MICCAI (2010) 10.1007/978-3-642-15745-5_61 20879352

[CR12] Nakamura T, Terano A (2008). Capsule endoscopy: past, present, and future. J. Gastroenterol..

[CR13] Newcombe, R.A, Izadi, S., Hilliges, O., Molyneaux, D., Kim, D., Davison, A.J., Kohi, P., Shotton, J., Hodges, S., Fitzgibbon, A.: Kinectfusion: real-time dense surface mapping and tracking. In: Mixed and Augmented Reality (ISMAR), 2011 10th IEEE International Symposium on, IEEE, pp. 127–136 (2011)

[CR14] Pan, G., Wang, L.: Swallowable wireless capsule endoscopy: progress and technical challenges. Gastroenterol Res Pract (2012) 10.1155/2012/841691 PMC3255457 22253621

[CR15] Ping-Sing T, Shah M (1994). Shape from shading using linear approximation. Image Vis. Comput..

[CR16] Qian, X., Sanchez, J., Sun, Y., Lin, B., Johnson, A.: Motion compensated slam for image guided surgery. Simultaneous tracking, 3D reconstruction and deforming point detection for stereoscope guided surgery (2013)

[CR17] Rubin JM, Xie H, Kim K, Weitzel WF, Emelianov SY, Aglyamov SR, Wakefield TW, Urquhart AG, ODonnell M (2006). Sonographic elasticity imaging of acute and chronic deep venous thrombosis in humans. J. Ultrasound Med..

[CR18] Sitti M, Ceylan H, Hu W, Giltinan J, Turan M, Yim S, Diller E (2015). Biomedical applications of untethered mobile milli/microrobots. Proc. IEEE.

[CR19] Son D, Yim S, Sitti M (2016). A 5-d localization method for a magnetically manipulated untethered robot using a 2-d array of hall-effect sensors. IEEE/ASME Trans. Mechatron..

[CR20] Stoyanov, D., Scarzanella, M.V., Pratt, P., Yang, G.-Z.: Real-time stereo reconstruction in robotically assisted minimally invasive surgery. In: International Conference on Medical Image Computing and Computer-Assisted Intervention, Springer, New York, pp. 275–282 (2010) 10.1007/978-3-642-15705-9_34 20879241

[CR22] Than TD, Alici G, Zhou H, Li W (2012). A review of localization systems for robotic endoscopic capsules. IEEE Trans. Biomed. Eng..

[CR23] Whelan, T., Leutenegger, S., Salas-Moreno, R.F., Glocker, B., Davison, A.J.: Elasticfusion: Dense slam without a pose graph. In: Robotics: Science and Systems, Vol. 11 (2015)

[CR24] Yim S, Goyal K, Sitti M (2013). Magnetically actuated soft capsule with the multimodal drug release function. IEEE/ASME Trans. Mechatron..

[CR25] Yim S, Gultepe E, Gracias DH, Sitti M (2014). Biopsy using a magnetic capsule endoscope carrying, releasing, and retrieving untethered microgrippers. IEEE Trans. Biomed. Eng..

